# Asserted Injurious Effects of the Manufacture of Lucifer Matches

**Published:** 1846-06

**Authors:** 


*Asserted Injurious Effects of the Manufacture of Lucifer Matches.*—Several German practitioners, and among others, M. Heyfelder, clinical professor of surgery at Erlangen, have published a number of cases of necrosis of the maxillary bones, which they attribute to the influence of the materials used in the manufacture of lucifer matches, the patients having thus been employed. M. Bricheteau, wishing to verify the accuracy of these statements, with reference to the asserted cause of the disease, has carefully visited all the manufactories in which chemical matches are made in Paris and the neighborhood. The results of M. Bricheteau's investigations do not appear to confirm the statements made in Germany. Among the two thousand workpeople, men and women, who are thus employed in or near Paris, it does not appear that a single well-authenticated case of necrosis of the maxillary bones has occurred. Three or four cases of disease of the maxillary are mentioned by him, but he states that the disease was either syphilitic, or existed before the patients had worked at the factory.

The manufacturers have remarked, that the vapors that result from the instantaneous combustion of the matches, which contain sulphurous acid, and phosphorous and phosphoric acid, make the workpeople cough, and that the coughs thus occasioned are more troublesome in winter than in summer, owing to the ventilation of the workshops not being then so good; but they have not observed any other symptoms.—*Jour. de Méd.—Bos. Med. & Surg. Jour.*

